# Correction: The rise and fall of the *Phytophthora infestans* lineage that triggered the Irish potato famine

**DOI:** 10.7554/eLife.01108

**Published:** 2013-07-02

**Authors:** Kentaro Yoshida, Verena J Schuenemann, Liliana M Cano, Marina Pais, Bagdevi Mishra, Rahul Sharma, Chirsta Lanz, Frank N Martin, Sophien Kamoun, Johannes Krause, Marco Thines, Detlef Weigel, Hernán A Burbano

Yoshida K, Schuenemann VJ, Cano LM, Pais M, Mishra B, Sharma R, Lanz C, Martin FN, Kamoun S, Krause J, Thines M, Weigel D, Burbano HA. 2013. The rise and fall of the *Phytophthora infestans* lineage that triggered the Irish potato famine. *eLife* **2**:e00731. doi: http://dx.doi.org/10.7554/eLife.00731. Published 28 May 2013

The article should have been published under the CC0 1.0 Universal (CC0 1.0) public domain dedication, not the Creative Commons Attribution 3.0 Unported License (CC BY 3.0).

The value corresponding to the sample EC3527 for the gene *Avr1* (Row 3, column 5) in Table 4 incorrectly gave a value of 100. The correct value is 0.

The article has been corrected accordingly.

