# Supplementary material for: The rise and fall of the Phytophthora infestans lineage that triggered the Irish potato famine
Source: eLife. 2013 May 28;2:e00731. doi: 10.7554/eLife.00731 (PMC3667578; doi:10.7554/eLife.00731)
Supplement: Table 5—source data 1. — Full-length sequences of deduced amino acid sequences of HERB-1 AVR1, AVR2, AVR3a and AVR4. DOI: http://dx.doi.org/10.7554/eLife.00731.022 [file elife00731s001.pdf]

> AVR1_ HERB1
MGLMHRVLLLATFALLCMHAKAAGFDHDKVPRTVERGGGARQLRTATMSDDEARVS
KLPSFIESFVKNRKIESWIQNKVTDDFVLSELKLVRLPGTSLADDPNFKLFQKFKIGGW
LEEKATTTKAWENLGLDSLPFDQVSK(I/T)DEFKTYTQYVT(V/A)LNKKASKLDIDQ
WHGLLSGGSPEELMAKAMILRTLGRDVLERRVMLGGHVVVPF

>AVR2_ HERB1
MRLAYIFAVMMAGALPYCNALHAAPGAKALKKIKTFPDFAAPSPKDGNRLLRRVDNG
EFEIEEERGFSLKDTLKKLNPIKAAVKAKDKAKEVTEKITDADWKKLVEHLKIKGDKR
S

> AVR3a_ HERB1
MRLAIMLSATAVAINFATCSAIDQTKVLVYGTPAHYIHDSAGRRLLRKNEENEETSEE
RAPNFNLANLNEEMFNVAALTKRADAKKLAKQLMGNDKLADAAYIWWQHNRVTLD
QIDTFLKLASRKTQGAKYNQIYNSYLMHLGLTGY

> AVR4_ HERB1
MRSLHILLVFTASLLASL(T/I)ESAKADSLARTVSVVDNVKVKSRFLRAQTDEKNEERA
TITLGDRVVSDKAATKDLLQQLLALGTPLEKVQKQFLNIPQMKTFAELSKHPNWKAL
DKYERMQWQKLKEGETLTFMRLGDRSYSKEKAQEQLLRWVAQKKPVESVYDDLQV
AGFAHNTVAARQNWRAYIMYDKWFTAASQMQRNPQQYAKFGTGYHSEQKTTEVFE
KWAMEGTHIKSVITTLKLNGKSASEMANNENFPALLKYVKLYLDFKPFRDLNAKSRL
QARRPIS

Heterozygous position are marked in red. The details are described in Table 5.
